# Systematic mapping on availability, extent and distribution of mental health research in Malawi

**DOI:** 10.4102/sajpsychiatry.v28i0.1810

**Published:** 2022-12-07

**Authors:** Genesis Chorwe-Sungani, Felix Chisoni, Ditress Nyirongo, Japhet Myaba, Anthony Sefasi, Jonas Sagawa, Grace Sibande, Costantine Chaima, Eluby N. Katola, Febbie Jamieson, Lucy Kululanga, Diana Jere

**Affiliations:** 1 Department of Mental Health, School of Nursing, Kamuzu University of Health Sciences, Blantyre, Malawi; 2 Library Department, Kamuzu University of Health Sciences, Blantyre, Malawi; 3 Department of Community Health, School of Nursing, Kamuzu University of Health Sciences, Blantyre, Malawi

**Keywords:** Malawi, systematic mapping, mental health, research, evidence

## Abstract

**Background:**

Mental health research is essential in the implementation of evidence-based interventions. This can be impeded by unavailability or limited access to local evidence in low- and middle-income countries (LMICs) such as Malawi.

**Aim:**

The aim of this systematic mapping was to describe the availability, extent and distribution of mental health research conducted in Malawi.

**Setting:**

The study was conducted at Kamuzu University of Health Sciences in Malawi.

**Methods:**

A systematic search of four electronic databases from inception to September 2021 was carried out. All published and unpublished mental health studies in all languages were eligible for inclusion. Studies were screened against inclusion and exclusion criteria, and data were extracted, analysed and presented in tables and as a narrative synthesis.

**Results:**

Cross-sectional studies (33.6%, *n* = 76) were found to be the most common study design for mental health research in Malawi. More studies were conducted on women (21.2%, *n* = 48) compared to men (1.3%, *n* = 3). Mental health research was concentrated in the southern region of the country (44.8%, *n* = 120) and in the three cities of Lilongwe (17.9%, *n* = 48), Blantyre (16.4%, *n* = 44) and Zomba (9.0%, *n* = 24).

**Conclusion:**

This systematic mapping suggests that there are few studies on mental health in Malawi which are not equally distributed across the country. There is a pressing need to conduct more mental health research using robust designs across disciplines.

**Contribution:**

Research on mental health is urgently needed to produce culturally acceptable data in Malawi.

## Introduction

Mental health research contributes to the provision of solutions for health problems and to the development of any country. However, unavailability of local evidence is often reported as a key barrier to improving health status and introducing new interventions in low- and middle-income countries (LMICs).^1^ It is documented that there is a dearth of research on effective mental health interventions in LMICs.^2^ This may be attributed to inadequate in-country research capacity which has led to limited availability of local evidence in LMICs,^1^ including Malawi. There is an emerging need for locally generated evidence to supplement the existing preponderance of evidence from high-income countries (HICs) to bring transformational health changes in LMICs.^1^

Mental health issues have been researched using various quantitative and qualitative research designs. Nevertheless, some research work in mental health that was conducted in Malawi remains as grey literature or is not published in peer-reviewed journals, and it may not be easily accessed by academics, clinicians and policymakers because most local universities only keep hardcopies of theses (not yet digitised) in the libraries. This may have a domino effect in decision-making because local evidence may not be readily obtainable^1^ to inform mental health interventions. However, accessibility of local evidence may be improved through systematic mapping of mental health research in Malawi. In this study, mental health research refers to any study that investigated biopsychosocial factors (how biological, psychological and social functioning interact), trends and social determinants in population mental health,^3^ treatment of mental disorders and psychosocial interventions.

Systematic mapping gathers, collates, describes and catalogues available evidence on a topic of interest.^4^ In addition, it provides searchable databases of studies together with descriptive and geographical information and patterns across research literature.^5^ In this systematic mapping, the authors intended to create a synopsis of the availability and distribution of evidence in the field of mental health across the country. It enabled them to compile the metadata of primary studies in mental health without synthesising the findings as is done in systematic reviews. Therefore, this article will describe the availability, extent and distribution of mental health research conducted in Malawi using systematic mapping.

### What is systematic mapping?

Systematic mapping is similar to systematic review in many ways.^5^ Both follow the same rigorous, objective and transparent process of capturing evidence relevant to a particular topic of interest.^4^ Systematic mapping aims to answer a broader question involving multiple interventions or exposures and/or multiple outcomes.^5,6^ The question can be open-framed or closed, and there is no limitation on research evidence that can be included.^4^

In systematic mapping, inclusion criteria may not be distinct; information describing a study and its method (metadata only) is extracted; critical appraisal is optional; and there is no synthesis of research findings.^5^ Furthermore, systematic mapping describes and catalogues relevant evidence on a topic of interest in searchable database and identifies knowledge gaps and clusters.^4,6^ It also makes implications for policy, practice and research.^4^ Systematic mapping serves as a resource to inform and commission systematic reviews, briefings and/or primary research).^4,6^

### Main question for the systematic mapping

The main question for this systematic mapping was: what is the availability, extent and distribution of mental health research conducted in Malawi?

### Aim of the systematic mapping

The aim of this systematic mapping was to describe the availability, extent and distribution of mental health research conducted in Malawi.

### Specific objectives of the systematic mapping

The following were the specific objectives for this systematic mapping:

- to document types of mental health research and locations that were conducted in Malawi
- to collate metadata for published research articles on mental health research in Malawi
- to identify knowledge gaps that may be suitable for primary mental health research in Malawi
- to provide digital object identifiers (DOIs) or Uniform Resource Locators (URLs) for locating mental health research that was conducted in Malawi
- to create a searchable database for mental health research that was conducted in Malawi.

## Research methods and design

### Criteria for considering studies for this systematic mapping

Selection of studies for inclusion in the systematic mapping was done by applying and extracting the PICOS criteria: participants (P), intervention (I), comparator (C), outcome measures (O), and study setting (S). In addition, all types of studies (quantitative, qualitative, mixed methods studies and systematic reviews) were considered for inclusion in the systematic mapping. Multicentre studies that were conducted in Malawi and other countries were also included. Studies conducted outside Malawi were excluded.

#### Types of participants

Participants were any group(s) of people or population(s). Categories considered included children, adolescents, youths, adults or elderly people living, working and accessing any services in various settings in Malawi.

#### Types of interventions

The authors included any studies reporting on prevalence of mental health problems; assessment, screening and diagnosis of mental health problems; treatment of mental health problems; psychosocial interventions; beliefs, perceptions and stigma about mental health; training related to mental health; mental health services; and ethical or legal issues.

#### Types of comparators

Where applicable, comparators included: (1) control group versus intervention group; (2) group with exposure versus group without exposure; and (3) index test versus reference test.

#### Outcome measures

The studies that were considered for inclusion in this systematic mapping were those that measured: prevalence of mental health problems and/or associated risk or protective factors; effectiveness of treatment of mental health problems or psychosocial interventions; knowledge, attitudes and skills related to mental health care; beliefs, perceptions, stigma and culture associated with mental health; training of mental health professionals and lay mental health workers; quality of mental health services; ethical and legal issues related to mental health; and accessibility, affordability and distribution of mental health services.

### Search methods for identification of studies

**FIGURE 1:**
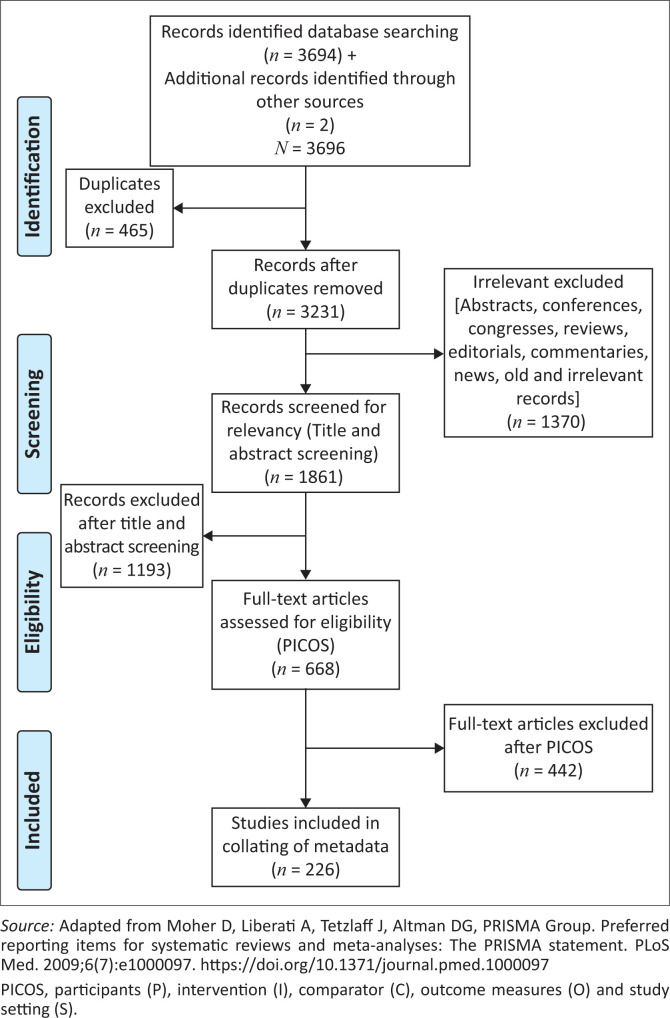
Flow diagram for selection of studies.

The search strategies for identifying primary studies incorporated the methodological component of the research recommended by Clapton et al. to establish a review team comprising all authors before systematic mapping was started.^6^ The team identified possible sources for relevant literature for them to make a comprehensive and unbiased search for this systematic mapping. Furthermore, this systematic mapping was conducted following four phases of searching and selecting appropriate papers, namely identification, screening, eligibility and inclusion (Figure 1).^7^

#### Electronic searches

The following electronic databases were searched for primary studies: (1) PubMed, Cumulative Index for Nursing and Allied Health Professions Literature (CINAHL), (2) Psych INFO and (3) ScienceDirect (Table 1).

**TABLE 1:** Search terms.

Database	Search terms used
PubMed	(((‘mental health’[MeSH Terms] OR (‘mental’[All Fields] AND ‘health’[All Fields]) OR ‘mental health’[All Fields] OR (‘mental disorders’[MeSH Terms] OR (‘mental’[All Fields] AND ‘disorders’[All Fields]) OR ‘mental disorders’[All Fields] OR (‘mental’[All Fields] AND ‘illness’[All Fields]) OR ‘mental illness’[All Fields]) OR (‘mental disorders’[MeSH Terms] OR (‘mental’[All Fields] AND ‘disorders’[All Fields]) OR ‘mental disorders’[All Fields] OR (‘psychiatric’[All Fields] AND ‘disorder’[All Fields]) OR ‘psychiatric disorder’[All Fields])) AND (‘therapeutics’[MeSH Terms] OR ‘therapeutics’[All Fields] OR ‘treatments’[All Fields] OR ‘therapy’[MeSH Subheading] OR ‘therapy’[All Fields] OR ‘treatment’[All Fields] OR ‘treatment s’[All Fields])) OR (‘therapeutics;[MeSH Terms] OR ‘therapeutics’[All Fields] OR ‘therapies’[All Fields] OR ‘therapy’[MeSH Subheading] OR ‘therapy’[All Fields] OR ‘therapy s’[All Fields] OR ‘therapies’[All Fields]) OR (‘psychosocial intervention’[MeSH Terms] OR (‘psychosocial’[All Fields] AND ‘intervention’[All Fields]) OR ‘psychosocial intervention’[All Fields])) AND (‘risk factors’[MeSH Terms] OR (‘risk’[All Fields] AND ‘factors’[All Fields]) OR ‘risk factors’[All Fields]) AND (‘epidemiology’[MeSH Subheading] OR ‘epidemiology’[All Fields] OR ‘prevalence’[All Fields] OR ‘prevalence’[MeSH Terms] OR ‘prevalence’[All Fields] OR ‘prevalences’[All Fields] OR ‘prevalence s’[All Fields] OR ‘prevalent’[All Fields] OR ‘prevalently’[All Fields] OR ‘prevalent’[All Fields]) AND (‘malawi’[MeSH Terms] OR ‘malawi’[All Fields] OR ‘malawi s’[All Fields])
CINAHL	TI mental health AND TI mental illness OR psychiatric disorder AND TI treatment OR therapy OR psychosocial intervention AND TI risk factors AND TI prevalence AND TI malawi AND LIMIT-TO (research article)
Psych INFO	Mental health AND psychiatric disorder AND risk factors AND prevalence AND therapy AND treatment AND psychosocial interventions AND malawi
ScienceDirect	ALL (‘mental health’ OR ‘psychiatric disorder’ OR ‘mental illness’) and ALL (treatment AND therapy OR psychosocial intervention) and ALL (risk factors) and ALL (prevalence) AND LIMIT-TO (topics, ‘malawi’)

CINAHL, Cumulative Index for Nursing and Allied Health Professions Literature.

The authors searched for published articles about the biopsychosocial factors (how biological, psychological and social functioning interact), trends and social determinants in population mental health treatment of mental disorders and psychosocial interventions in Malawi. An initial search firstly involved a search across all databases that were included using all identified keywords and index terms. Secondly, additional studies were sought from the reference list of all identified articles. The systematic mapping included studies that were published in English. The first author, together with an information scientist (F.C.), constructed the following search terms that were initially used to conduct a search: (Mental health OR Mental illness Or Mental Disorders OR Mental Health problems) AND (prevalence OR treatment OR psychosocial intervention) AND (Malawi OR ‘Name of specific district’). The final search for each database was done using search terms that are presented in Table 1.

#### Searching other resources

Reference lists of the included studies were hand searched by the first author (G.C.-S.). Experts with a master’s or doctorate degree in mental health nursing, psychology and psychiatry from within Malawi and around the world who had published at least one mental health research conducted in Malawi were also contacted. These experts were requested to provide names of persons or institutions that might have expert knowledge regarding relevant publications for mental health in Malawi.

### Data collection

Studies retrieved from the final search were uploaded onto the Reference Manager in Endnote (Clarivate Plc, London, United Kingdom) and screened for duplicates. The inclusion and exclusion criteria were then independently applied by three authors (G.C.-S.).

### Data analysis

#### Synthesis of evidence

In this systematic mapping, a synthesis of trends in the literature, knowledge gaps and clusters identified was carried out without synthesising study results.^4^ The following coding variables were tabulated and used to extract metadata: (1) full reference including URL and DOIs; (2) year of publication; (3) publication type; (4) journal type; language; (5) study location (district or city); (6) data type; (7) study design; (8) population(s); (9) and mental health issue researched. Descriptive statistics were used to summarise the data.

#### Outputs

The systematic mapping process achieved the following outputs: the status of local evidence on mental health issues was described; the geographical distribution of mental health research conducted in Malawi was described; the knowledge gaps related to mental health issues in Malawi were identified; and a searchable list of studies on mental health issues in Malawi was created (Online Appendix 1).

### Ethical considerations

No ethical clearance was needed and/or required for this study as it involved review of data from primary studies which had previously received ethics approval.

## Results

### Review process and results

The electronic search yielded 3694 published records from Pubmed, CINAHL, PsychINFO and ScienceDirect, (Figure 1). Two articles were sourced from authors, resulting in a total of 3696 records (Figure 1). A total of 465 duplicates were removed, resulting in 3231 records that were scrutinised. A further 1370 records were removed because they were irrelevant, conferences, congresses, editorials, commentaries, reviews and news, resulting in 1861 articles that were assessed for relevancy. A further 1193 articles were excluded after reviewers screened their titles and abstracts for relevancy, resulting in 668 that were scrutinised using the PICOS criteria. The application of the PICOS criteria resulted in excluding a further 442 articles, leaving 226 articles that were included in this systematic mapping (Online Appendix 1). The reviewers’ ratings for inclusion or exclusion of studies agreed with a kappa = 0.94.

### Findings from studies for inclusion in the systematic mapping (*n* = 226)

Of the 226 articles that were included in this systematic mapping, most of them were published in English (*n* = 225) between 1988 and 2021. There was only one article that was published in Norwegian (*n* = 1).

#### Status of local evidence on mental health issues

**Publications on mental health research:** The decadal categorisation of publications was used for easy categorisation of mental health research in this study. This systematic mapping showed that there were few research studies on mental health issues in Malawi that were published before the year 2000 (Table 2). However, the mapping also revealed that there has been a plethora of scientific publications on mental health issues (78.3%, *n* = 177) in the country since 2011. This systematic mapping further found that many research studies that were conducted in Malawi were published in peer-reviewed journals (90.7%, *n* = 205) (Table 2). The mapping also showed that some studies were available online in the form of dissertations or theses (6.6%, *n* = 15).

**TABLE 2:** Publications on mental health research (*n* = 226).

Item	*n*	%
**Publication type**		
Book article	2	0.9
Dissertation or thesis	15	6.6
Journal article	205	90.7
Preprint	1	0.4
Report	3	1.3
**Year of publication**		
1988–1999	14	6.2
2000–2010	35	15.5
2011–2021	177	78.3

**Methodologies used to conduct mental health research:** The systematic mapping revealed that quantitative studies (75.2%, *n* = 170) dominated mental health research in Malawi, followed by qualitative methods (18.6%, *n* = 42). Furthermore, some mental health research (6.2%, *n* = 14) had used mixed methods.

**Groups of participants in mental health research:** This systematic mapping revealed that people without known mental or physical problems (71.7%, *n* = 162) were the group that participated in most mental health research in Malawi (Table 3).

**TABLE 3:** Groups of participants in mental health research (*n* = 226).

Group of participants	*n*	%
Healthcare providers	18	8
Patients with physical problems	19	8.4
Patients with mental disorders	27	11.9
People without known mental or physical problems	162	71.7

**Areas covered in mental health research:** This systematic mapping showed that psychosocial well-being and problems (47.3%, *n* = 107) was the area that was most covered in mental health research in Malawi (Table 4). There was less than 10.0% of mental health research that focused on treatment and interventions in the country.

**TABLE 4:** Areas covered in mental health research (*n* = 226).

Mental health issue	*n*	%
Mental disorders	80	35.4
Psychosocial well-being and problems	107	47.3
Treatment and intervention	21	9.3
Mental health services and training	11	4.9
HIV and mental health	7	3.1

#### Geographical distribution of mental health research in Malawi

**FIGURE 2:**
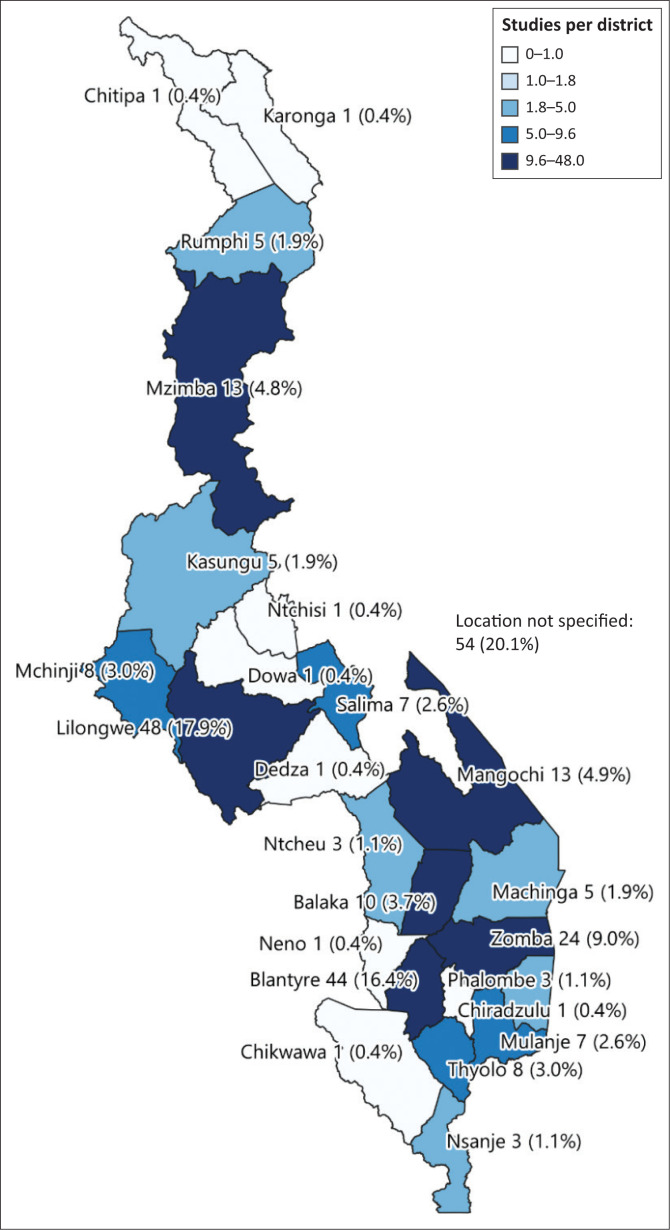
Geographical distribution of mental health research by district (*n* = 268).

**FIGURE 3:**
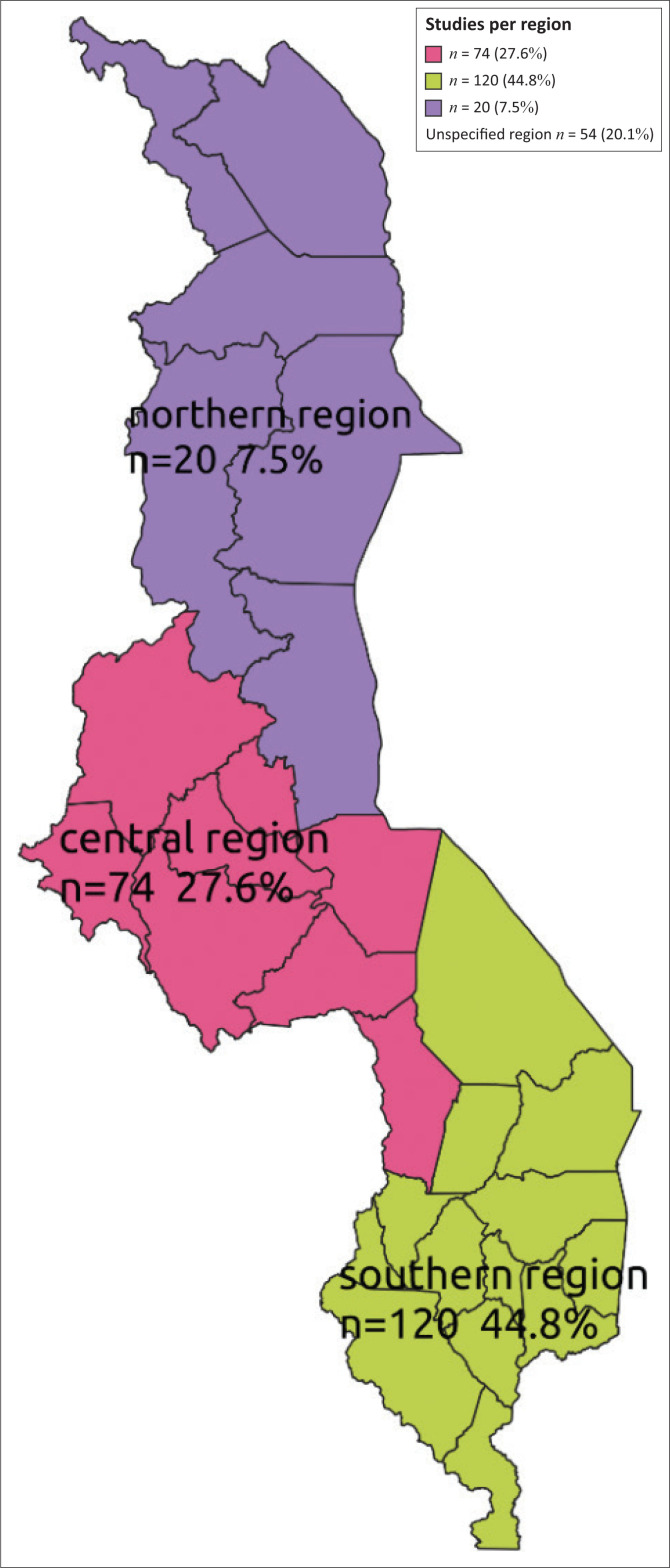
Geographical distribution of mental health research by region (*n* = 268).

This systematic mapping found that research on mental health issues was concentrated in the three cities of Lilongwe (17.9%, *n* = 48), Blantyre (16.4%, *n* = 44) and Zomba (9.0%, *n* = 24), with minimal studies conducted in other districts across the country (Figure 2). Some studies were conducted in multiple districts (42), increasing *n* to 268 when each district was counted alone against a particular study (Figure 2). However, no published studies on mental health issues from Likoma, Nkhotakota and Mwanza districts were found. Furthermore, the systematic mapping showed that many studies (44.8%, *n* = 120) were conducted in the southern region of the country (Figure 3).

## Discussion

This first systematic mapping on mental health research has showed the availability, distribution and status of local evidence in Malawi. The systematic mapping suggests that mental health is relatively under-researched. This is corroborated by Read and Doku,^8^ who asserted that mental health research in Ghana was limited in both quantity and quality. There is limited investment in mental health research (less than 1% of gross domestic product [GDP]) in LMICs^9^ such as Malawi, which has resulted in insufficient local evidence that can inform mental health practice. There is lack of local evidence from randomised controlled trials to show whether a certain mental health intervention works in Malawi.

This systematic mapping has shown that quantitative studies dominated research that was conducted in Malawi. It is possible that quantitative methods are used more compared to qualitative methods because the questions being asked require quantitative methods to answer. Some quantitative designs (cross-sectional designs) may be preferred to answer questions because they are also easier to develop, easier to assess what a researcher would like to measure and easier to administer, as they require less training, take less time and can be used with bigger samples.^10^ Furthermore, this may also partly be explained by limited and unequal distribution of mental health research capacity in LMICs^9^ such as Malawi. This may be the case in Malawi, where mental health specialists rarely conduct mental health research using mixed methods designs (6.2%, *n* = 14). It is necessary that all health professionals must be trained to conduct clinically based research^8^ using a variety of relevant and robust research methods. Since 2010, there has been an increase in mental health research from master’s or doctoral students in Malawi. However, many studies are concentrated in the southern region of the country. This may be explained by the fact that the main referral mental hospital is located in the southern region of the country (Zomba), which makes it easy for researchers to access target populations of clients with mental disorders. Furthermore, the studies are conducted around this facility and cities because of the availability of the specialised mental health services.

The sparse distribution of research in other regions of the country may imply that the available evidence may not be representative for the country. There is a need to conduct mental health research in the central and northern regions of the country. In addition, there is a need for future studies to use representative samples from across the country to ensure that cross-cultural and culturally relevant evidence is generated locally. This will help to produce the much-needed evidence to understand the complex mental disorders in the country. For instance, this systematic mapping showed that mental disorders constituted only one third of mental health research (35.4%, *n* = 80) that was conducted in the country. This may be attributed to challenges which researchers encounter to recruit clients with mental disorders in mental health research.^11^ Nevertheless, some mental disorders such as psychosis have been significantly researched worldwide because of their devastating nature, poor prognosis and reduced remission.^12^ In addition, literature also indicated that depression is one of the most intensely researched mental disorders^13^ globally.

Despite limitations mentioned before, psychosocial well-being and problems covered nearly half of the mental health research (47.3%, *n* = 107) that was conducted in Malawi. This can be explained by literature which showed crosscutting efforts from stakeholders who are interested in implementing evidence-based interventions for dealing with psychosocial problems such as violence to uphold the human rights of women and children.^14,15,16,17^ There is a significant amount of mental health research on psychosocial well-being and problems that is conducted by non–mental health specialists, while mental health research on mental disorders is mainly conducted by mental health specialists.

### Study limitations

This systematic mapping is limited because these sources of data may not have been exhaustive, and some relevant search terms may have been left out. It is possible that this systematic mapping did not identify all published studies and grey literature through these search methods. However, this systematic mapping can be updated in future to include studies that were not accessible during the data collection period and those studies that will be conducted in future.

## Conclusion

This systematic mapping has provided important insights into the research of mental health issues in Malawi and suggested directions for future research. There is an urgent need for mental health research using robust study designs and representative samples to generate useful, local and culturally acceptable evidence across disciplines. Based on this systematic mapping, it is suggested that population-based epidemiological studies of mental disorders, research on mental disorders and studies on psychosocial interventions should be prioritised in Malawi.

## References

[1] Lahariya C. Introducing healthcare in low-resource settings. Healthcare Low Resour Sett. 2013;1(1):1. 10.4081/hls.2013.e1

[2] Herba CM, Glover V, Ramchandani PG, Rondon MB. Maternal depression and mental health in early childhood: An examination of underlying mechanisms in low-income and middle-income countries. Lancet Psychiatry. 2017;3(10):983–992. 10.1016/S2215-0366(16)30148-1 27650772

[3] Bhakta S. The importance of mental health research and evaluation. Mental Health First Aid [serial on the Internet]. 2021 [cited 2021 Aug 5]. Available from: https://www.mentalhealthfirstaid.org/external/2021/04/the-importance-of-mental-health-research-and-evaluation/.

[4] James KL, Randall NP, Haddaway NR. A methodology for systematic mapping in environmental sciences. Environ Evid. 2016;5(1):7. 10.1186/s13750-016-0059-6

[5] Haddaway NR, Bernes C, Jonsson B-G, Hedlund K. The benefits of systematic mapping to evidence-based environmental management. Ambio. 2016;45(5):613–620. 10.1007/s13280-016-0773-x 26984257 PMC4980318

[6] Clapton J, Rutter D, Sharif N. SCIE systematic mapping guidance. London: SCIE; 2009.

[7] Moher D, Liberati A, Tetzlaff J, Altman DG, PRISMA Group. Preferred reporting items for systematic reviews and meta-analyses: The PRISMA statement. PLoS Med. 2009;6(7):e1000097. 10.1371/journal.pmed.1000097 19621072 PMC2707599

[8] Read UM, Doku V. Mental health research in Ghana: A literature review. Ghana Med J. 2012;46(2):29–38. 23661815 PMC3645145

[9] Razzouk D, Sharan P, Gallo C, et al. Scarcity and inequity of mental health research resources in low-and-middle income countries: A global survey. Health Policy. 2010;94(3):211–220. 10.1016/j.healthpol.2009.09.009 19846235

[10] Kielmann K, Cataldo F, Seeley J. Introduction to qualitative research methodology: A training manual. pp. 1–4. London: Department for International Development (DfID); 2012.

[11] Bucci S, Butcher I, Hartley S, Neil ST, Mulligan J, Haddock G. Barriers and facilitators to recruitment in mental health services: Care coordinators’ expectations and experience of referring to a psychosis research trial. Psychol Psychother Theory Res Pract. 2015;88(3):335–350. 10.1111/papt.12042 25257960

[12] Adjorlolo S, Setordzi M. Psychosis in adolescents in Africa: A scoping review for current understanding and future directions. Cogent Psychol. 2021;8(1):1949173. 10.1080/23311908.2021.1949173

[13] Witcomb GL, Bouman WP, Claes L, Brewin N, Crawford JR, Arcelus J. Levels of depression in transgender people and its predictors: Results of a large matched control study with transgender people accessing clinical services. J Affect Disord. 2018;235:308–315. 10.1016/j.jad.2018.02.051 29665513

[14] Ameli V, Meinck F, Munthali A, Ushie B, Langhaug L. Associations between adolescent experiences of violence in Malawi and gender-based attitudes, internalizing, and externalizing behaviors. Child Abuse Negl. 2017;67:305–314. 10.1016/j.chiabu.2017.02.027 28327416

[15] Banks LM, Kelly SA, Kyegombe N, Kuper H, Devries K. ‘If he could speak, he would be able to point out who does those things to him’: Experiences of violence and access to child protection among children with disabilities in Uganda and Malawi. PLoS One. 2017;12(9):e0183736. 10.1371/journal.pone.0183736 28926598 PMC5604937

[16] Mkandawire-Valhmu L, Bauer WS, Stevens PE, et al. Rural Malawian women’s resistance to systematic oppression, violence, and abuse by their husbands. J Interpers Violence. 2020;35(1–2):268–293. 10.1177/0886260516682518 29294622

[17] Palermo T, Pereira A, Neijhoft N, et al. Risk factors for childhood violence and polyvictimization: A cross-country analysis from three regions. Child Abuse Negl. 2019;88:348–361. 10.1016/j.chiabu.2018.10.012 30554126

